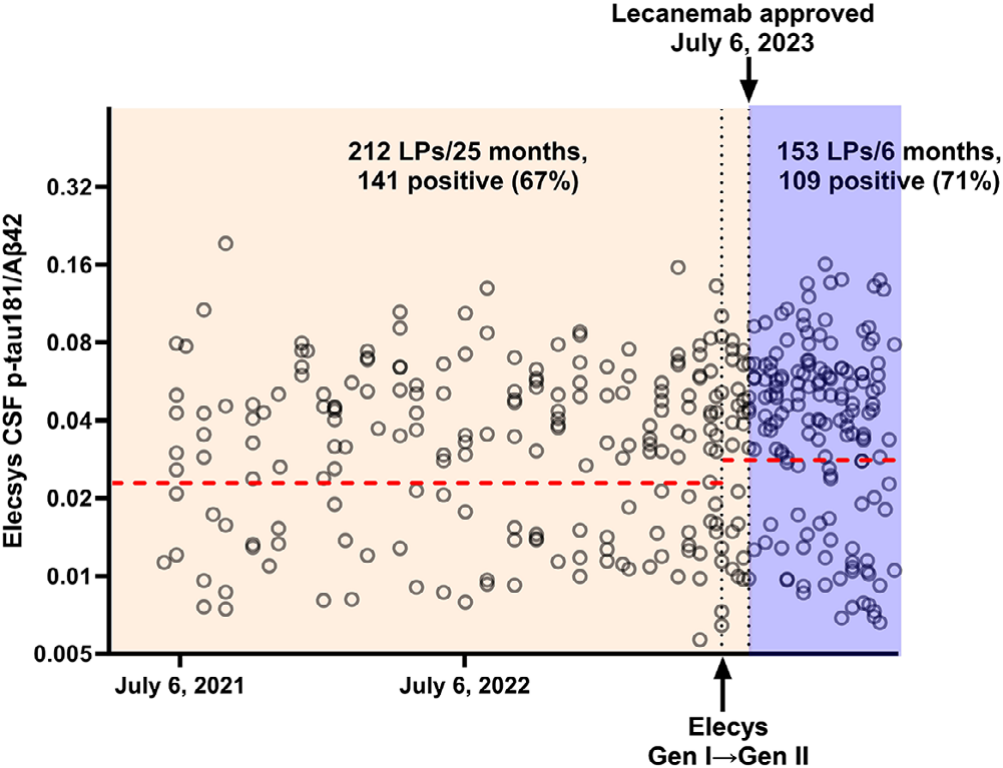# Pragmatic considerations in clinical biomarker testing for Alzheimer disease

**DOI:** 10.1002/alz.088362

**Published:** 2025-01-09

**Authors:** Suzanne E. Schindler, Madeline Paczynski, Dawn Ellington, B. Joy Snider

**Affiliations:** ^1^ Knight Alzheimer Disease Research Center, St. Louis, MO USA; ^2^ Knight Alzheimer Disease Research Center, Saint Louis, MO USA

## Abstract

**Background:**

With the traditional FDA approval of lecanemab for treatment of early symptomatic Alzheimer disease (AD), there has been a surge in demand for clinical AD biomarker testing. While amyloid PET, cerebrospinal fluid (CSF) biomarkers, and AD blood tests are all clinically available, each have different benefits and limitations affecting their real‐world use.

**Methods:**

Pragmatic issues affecting usage of different AD biomarker testing modalities have been experienced in a large specialty memory clinic in St. Louis, MO, USA, that has been scaling up clinical AD biomarker testing, particularly in patients who are candidates for lecanemab treatment.

**Results:**

The high and/or uncertain cost of amyloid PET has limited its use to a small number of patients. CSF biomarkers are the major modality used for AD biomarker testing. The number of lumbar punctures (LPs) has increased from ∼5/month in 2018‐2022 to ∼25/month after the approval of lecanemab. Most patients are willing to undergo LP, the procedure is tolerated well, the cost to patients is reasonable, and insurers are accepting a positive CSF test as sufficient evidence of amyloid pathology for initiation of lecanemab. However, reimbursement for LP is inadequate and LP clinics are burdensome to staff and patients. Approximately 200 AD blood tests have been performed in clinic patients over the past three years. Factors limiting use of AD blood tests include the cost to patients and private insurers not accepting AD blood tests for initiation of lecanemab (Medicare is accepting AD blood tests). More broadly, clinical use of AD blood tests has been complicated by the availability of tests with varying levels of accuracy and interpretability.

**Conclusions:**

Because the major indication for clinical AD biomarker testing is now confirmation of amyloid pathology in patients who are candidates for lecanemab, insurance decisions about which biomarkers are acceptable are driving which AD biomarker modalities are performed. When high accuracy AD blood tests are reimbursed by insurance and accepted as evidence of amyloid pathology, it is likely they will become the dominant modality of AD biomarker testing due to lower burden on staff and patients.